# A Human Cell Line Model for Interferon-α Driven Dendritic Cell Differentiation

**DOI:** 10.1371/journal.pone.0135219

**Published:** 2015-08-07

**Authors:** Jurjen M. Ruben, Lindy L. Visser, Kimberley M. Heinhuis, Tom O’Toole, Hetty J. Bontkes, Theresia M. Westers, Gert J. Ossenkoppele, Tanja D. de Gruijl, Arjan A. van de Loosdrecht

**Affiliations:** 1 Dept of Hematology, VU University Medical Center, Cancer Center Amsterdam, De Boelelaan 1117, 1081HV Amsterdam, The Netherlands; 2 Dept of Molecular Cell Biology and Immunology, VU University Medical Center, Cancer Center Amsterdam, De Boelelaan 1117, 1081HV Amsterdam, The Netherlands; 3 Dept of Medical Oncology, VU University medical center-Cancer Center Amsterdam, De Boelelaan 1117, 1081 HV Amsterdam, The Netherlands; University of Cape Town, SOUTH AFRICA

## Abstract

The CD34^+^ MUTZ-3 acute myeloid leukemia cell line has been used as a dendritic cell (DC) differentiation model. This cell line can be cultured into Langerhans cell (LC) or interstitial DC-like cells using the same cytokine cocktails used for the differentiation of their primary counterparts. Currently, there is an increasing interest in the study and clinical application of DC generated in the presence of IFNα, as these IFNα-DC produce high levels of inflammatory cytokines and have been suggested to be more potent in their ability to cross-present protein antigens, as compared to the more commonly used IL-4-DC. Here, we report on the generation of IFNα-induced MUTZ-DC. We show that IFNα MUTZ-DC morphologically and phenotypically display characteristic DC features and are functionally equivalent to “classic” IL-4 MUTZ-DC. IFNα MUTZ-DC ingest exogenous antigens and can subsequently cross-present HLA class-I restricted epitopes to specific CD8^+^ T cells. Importantly, mature IFNα MUTZ-DC express CCR7, migrate in response to CCL21, and are capable of priming naïve antigen-specific CD8^+^ T cells. In conclusion, we show that the MUTZ-3 cell line offers a viable and sustainable model system to study IFNα driven DC development and functionality.

## Introduction

Dendritic cells (DC) have been exploited for anti-cancer vaccination strategies since their successful generation *in vitro*, some two decades ago [1,2]. Since DC are present in blood at low numbers [3], studies have used DC generated from either monocytes (monocyte-derived dendritic cells; MoDC), or from CD34^+^ hematopoietic progenitor cells [4–6]. Most frequently MoDC are used, for which monocytes are isolated from blood and cultured in the presence of the differentiation inducing cytokines GM-CSF and IL-4, for 3 to 5 days [7,8]. Although differentiating MoDC using IL-4 and GM-CSF(IL-4 MoDC) is still the gold standard, recent reports suggest that MoDC cultured in the presence of GM-CSF and the type-I interferon interferon-α (IFNα MoDC) have potential benefits over conventional IL-4 MoDC. IFNα MoDC are for instance reported to have an increased antigen uptake potential and retention time, as well as an increased capacity to cross-present synthetic long peptides (SLP) [9–11]. However, we found IL-4 MoDC to be more potent in their ability to cross-prime CD8^+^ T cells against epitopes derived from an apoptotic cell-associated source, as compared to IFNα MoDC [12]. Monocytic and CD34^+^ progenitor cells provide solid model systems for generating DC, but generating DC from these progenitors for research purposes introduces donor variability and provides relatively short-lived sources of DC and their progenitors. Therefore, multiple cell lines have been explored as models for DC differentiation [13,14]. One such cell line is the cytokine-dependent CD34^+^ MUTZ-3 monocytic acute myeloid leukemia (AML) cell line, which has previously been used as a sustainable model to study human DC differentiation and maturation (interstitial DC-like [IDC] and Langerhans cells [LC]) *in vitro* [15–18]. MUTZ-3 progenitor cells can be differentiated into IDC (MUTZ-DC) by stimulation with GM-CSF, TNFα and IL-4, similar to the differentiation of monocytes into monocyte-derived dendritic cell (MoDC) or to LC-like cells by exposure to GM-CSF, TNFα, and TGFβ. Importantly, phenotypically and functionally these MUTZ-DC and–LC fully resemble and behave like their physiological counterparts [14,19]. Moreover, we have recently reported the rapid 3-day generation of MUTZ-DC, by exposure to low concentrations of the anthracyclin mitoxantrone, supplemented with GM-CSF and IL-4 [20]. The MUTZ-3 platform is therefore a convenient alternative to monocytes and primary CD34^+^ progenitor cells for the generation of human DC-like cells. An added advantage is its long-term sustainability, allowing for standardized culture and the possibility of generating stable transfectants for mechanistic, functional and developmental studies. Since there is growing interest in IFNα DC as vaccine vehicles, due to their reported superior CD8^+^ T cell (cross-)priming ability. For these reasons, we tested the possibility to rapidly differentiate MUTZ-3 progenitors into functional MUTZ-3 DC under the influence of GM-CSF, IFNα and mitoxantrone, and assessed their phenotype and functionality in direct comparison to similarly generated classic IL-4 MUTZ-DC. We show that the MUTZ-3 cell line can be used as a platform to study IFNα driven DC differentiation.

## Materials and Methods

### MUTZ-3 culture and MUTZ-DC differentiation

MUTZ-3 (Deutsche Sammlung von Mikroorganismen und Zellkulturen [DSMZ], Braunschweig, Germany) was maintained by seeding 2*10^5^ progenitor cells twice weekly in fresh MEM-α medium (Lonza, Breda, The Netherlands), supplemented with 10% fetal calf serum (FCS), 100 IU/ml penicillin, 100 μg/ml streptomycin (all Gibco, Paisley, UK) (further referred to as complete MEM-α), and 25 IU/ml GM-CSF (Peprotech, The Netherlands). MUTZ-DC were induced by culturing 3*10^5^/ml MUTZ-3 progenitor cells in complete MEM-α, supplemented with 500 IU/ml GM-CSF(Peprotech), 240 IU/ml TNFα (Sanquin, Amsterdam, The Netherlands), 2nM Mitoxantrone (Sigma-Aldrich, Zwijndrecht, The Netherlands), and either 10 ng/ml IL-4 (Peprotech) for inducing IL-4 MUTZ-DC, or 1000 IU/ml IFNα (Peprotech) for the induction of IFNα MUTZ-DC. After 3 days the MUTZ-DC were harvested, counted and either used for subsequent experiments (immature MUTZ-DC), or maturated by seeding 3.12*10^5^/ml MUTZ-DC in DC CellGro medium (Cell Genix, Freiburg, Germany), supplemented with 2400 IU/ml TNFα (Sanquin), 750 IU/ml IL-1β (Sanquin) and 1 μg/ml PGE_2_ (Sigma-Aldrich). After 24 hours, MUTZ-DC were harvested and used for subsequent experiments.

The MUTZ-DC phenotype was analyzed directly after differentiation (3 days), or after subsequent maturation, by analyzing the expression of CD1a-FITC (Dako Cytomation, Heverlee, Belgium), CD14-FITC, CD86-PE, CD83-PE, DC-SIGN-FITC (BD Biosciences, Breda, The Netherlands), CD40-FITC (Beckman Coulter, Woerden, The Netherlands), and an unlabeled CCR7 IgM antibody (BD Biosciences), followed by PE-conjugated goat anti-mouse IgM (Beckman Coulter), using flow cytometry (LSRFortessa, BD Biosciences). The corresponding isotype control antibodies were obtained from BD Biosciences.

The mean fluorescence index was calculated by dividing the mean fluorescence intensity of the antigen mAb staining with the mean fluorescence intensity of the corresponding isotype control.

### MUTZ-DC antigen uptake, migration and CD40 ligation

Apoptotic blebs were isolated from HL60 AML cells, as described previously [21]. In short, apoptosis was induced by heat-shock followed by γ-radiation, after which the cell suspension was harvested after 72 hours. The apoptotic cells were removed by centrifugation at 600 x *g*, and the resulting supernatant was spun down at 4,000 x *g*, pelleting the blebs. The uptake of apoptotic blebs by MUTZ-DC was quantified by co-culturing CFSE (Invitrogen, Breda, The Netherlands) labeled MUTZ-DC with PKH26 (Sigma-Aldrich) apoptotic blebs, and analyzing the percentage of MUTZ-DC that were PKH26 positive using flow cytometry (LSRFortessa, BD Biosciences). In related experiments, lysosomes of MUTZ-DC were labeled using Lyso Tracker RedDND-99 (Life Technologies, Bleiswijk, The Netherlands). The percentage of CFSE^+^ MUTZ-DC wherein PKH26^+^ apoptotic blebs and Lyso Tracker Red DND-99^+^ lysosomes co-localized was determined by ImageStream (Merck Millipore). Single cells, focused and CFSE positive, were scored for colocalization of the PKH26 and Lyso Tracker Red, using the Similarity Feature in the IDEAS (Merck Millipore) analysis software. Moreover, immature MUTZ-DC were visualized using the bright field channel of the ImageStream.

Pinocytosis and receptor-mediated antigen uptake was assessed by culturing 5*10^4^ immature MUTZ-DC in the presence of respectively Lucifer Yellow (2 μg/ml, Sigma-Aldrich), or Dextran-FITC (2 μg/ml, Sigma-Aldrich). After 1 hour the uptake was analyzed using flow cytometry (LSRFortessa) and plotted by dividing the mean fluorescence intensity of MUTZ-DC that were co-cultured with either Lucifer yellow, or Dextran-FITC, with that of unloaded MUTZ-DC.

The capacity of MUTZ-DC to migrate towards the lymph node homing chemokine CCL21 (Invitrogen, Carlsbad, USA) was analyzed using a transwell migration assay with a 5 μm pore size. 1*10^5^ immature or matured MUTZ-DC were added to the upper compartment of a transwell (Corning Costar, Landsmeer, The Netherlands) after which the migration towards the lower compartment (a 24-wells cell culture plate) was analyzed. The number of spontaneously migrated MUTZ-DC (the lower compartment containing plain DC CellGro medium) was subtracted from the number of MUTZ-DC that had actively migrated towards the lower compartment that was supplemented with 250 U/ml CCL21.

The production of pro-inflammatory cytokines by MUTZ-DC was analyzed by co-culture of 4*10^4^MUTZ-DC with the CD40 ligand expressing and irradiated cell line J558, in the presence of 1000 U/ml IFNγ (Sanquin, Amsterdam, The Netherlands), at a 1:1 ratio. After 24 hours, the supernatant was harvested and frozen for further analysis. The supernatants were thawed and analyzed for cytokines using an inflammatory cytokine bead array kit (CBA; BD Biosciences).

### Mixed leukocyte reaction

After differentiation, MUTZ-DC were cultured in the presence or absence of a maturation-inducing cytokine cocktail (TNFα, IL-1β and PGE_2_) for 24 hours, and subsequently harvested and co-cultured with CFSE-labeled CD14 depleted allogeneic peripheral blood leukocytes (PBL) in a mixed leukocyte reaction (MLR). After 6 days, the cells were harvested and labeled with CD3, CD4 and CD8 (all BD Biosciences), after which the CFSE dilution on CD4^+^ and CD8^+^ T cells was analyzed as a measure for proliferation, using flow cytometry (LSRFortessa, BD Biosciences). The coculture supernatant (harvested at day 6) was frozen and stored for analysis of cytokines, which was performed using a T_H_1/T_H_2/T_H_17 CBA kit (BD Biosciences) following manufacturers’ instructions.

### Cross-presentation

1*10^5^ MUTZ-DC were cultured overnight in the presence of the maturation-inducing cytokine cocktail with different concentrations of a 25-mer MART-1 synthetic long peptide (SLP) (aa16-40L), which was synthesized in-house, as described previously [22]. Next, loaded MUTZ-DC were harvested and cultured with a MART-1_aa26-35_ recognizing cytotoxic T cell line (>95% pure by dextramer binding analysis) for 5 hours, in the presence of 1 μl/ml of the golgi inhibitor GolgiStop (BD Biosciences). The cells were subsequently washed and stained with MART-1_aa26-35_ dextramer (Immudex, Copenhagen, Denmark) for 15 minutes, followed by 15 minutes of labeling with CD3 Horizon and CD8 (BD Biosciences). After washing, the cells were fixed and permeabilized using BD Cytofix/Cytoperm solution (BD Biosciences), following manufacturer’s protocol. The intracellular IFNγ levels were determined by staining for 30 minutes at 4°C with anti-IFNγ-PE (BD Biosciences), after which the cells were washed and IFNγ production was analyzed using flow cytometry (LSRFortessa, BD Biosciences), as a measure of activation.

### CD8^+^ T cell priming, sorting and avidity

Mature MUTZ-DC were harvested and resuspended in CellGro containing 1 μg/ml MART-1_aa26-35L_ (a peptide (ALGIGILTV) with a higher affinity for HLA-A2 [19]) peptide and 3 μg/ml β2-microglobulin for 2 hours at 37°C. Next, 1*10^5^ exogenously loaded MUTZ-DC were washed and resuspended in Yssels medium [23], and co-cultured with 1*10^6^ irradiated CD14^-^/CD8^-^ PBL and HLA-A2 matched 1*10^6^ CD8^+^ T cells in a total volume of 2 ml Yssels medium, as described previously [21]. After 10 days of culture the CD8^+^ T cells were restimulated by removing half of the medium, and adding 1*10^5^ freshly cultured and loaded MUTZ-DC in 1 ml Yssels to each well. The CD8^+^ T cells were restimulated weekly, and the percentage of MART-1 specific CD8^+^ T cells was analyzed by labeling the cells with a MART-1 dextramer, and visualized using flow cytometry (LSRFortessa). After three restimulations, the CD8^+^ T cells staining positive for the MART-1 dextramer were sorted using a BD FACSAria III cell sorter (BD Biosciences), and polyclonally expanded by stimulating the sorted cells weekly (4 weeks maximum) with 1*10^6^ irradiated peripheral blood mononuclear cells and 1*10^5^ irradiated JY cells, supplemented with 1 μg/ml phytohaemagglutanin (PHA), and 20 U/ml IL-7 (Sanquin). Next, T cell avidity was determined by loading JY cells with titrated concentrations of the of MART-1_aa26-35L_ peptide (ranging from 10 μM to 100 pM), and subsequently co-culturing them with primed and enriched CD8^+^ T cells for 5 hours in the presence of 1 μl/ml of GolgiStop (BD Biosciences). The cells were subsequently washed and stained with MART-1_aa26-35_ dextramer (Immudex, Denmark) for 15 minutes, followed by 15 minutes of labeling with CD3 and CD8 (BD Biosciences). After washing, the cells were fixed and permeabilized using BD Cytofix/Cytoperm solution (BD Biosciences), following manufacturer’s protocol. The intracellular IFNγ levels were determined as a measure of activation, as described above.

### Statistical analysis

Statistical analysis was performed with GraphPad Prism version 5 for Windows (GraphPad Software Inc.), using a paired two-tailed Student’s *t*-test. P-values ≤ 0.05 were regarded as significant.

## Results

### IFNα MUTZ-DC display a typical DC phenotype after differentiation and maturation

**Fig 1:**
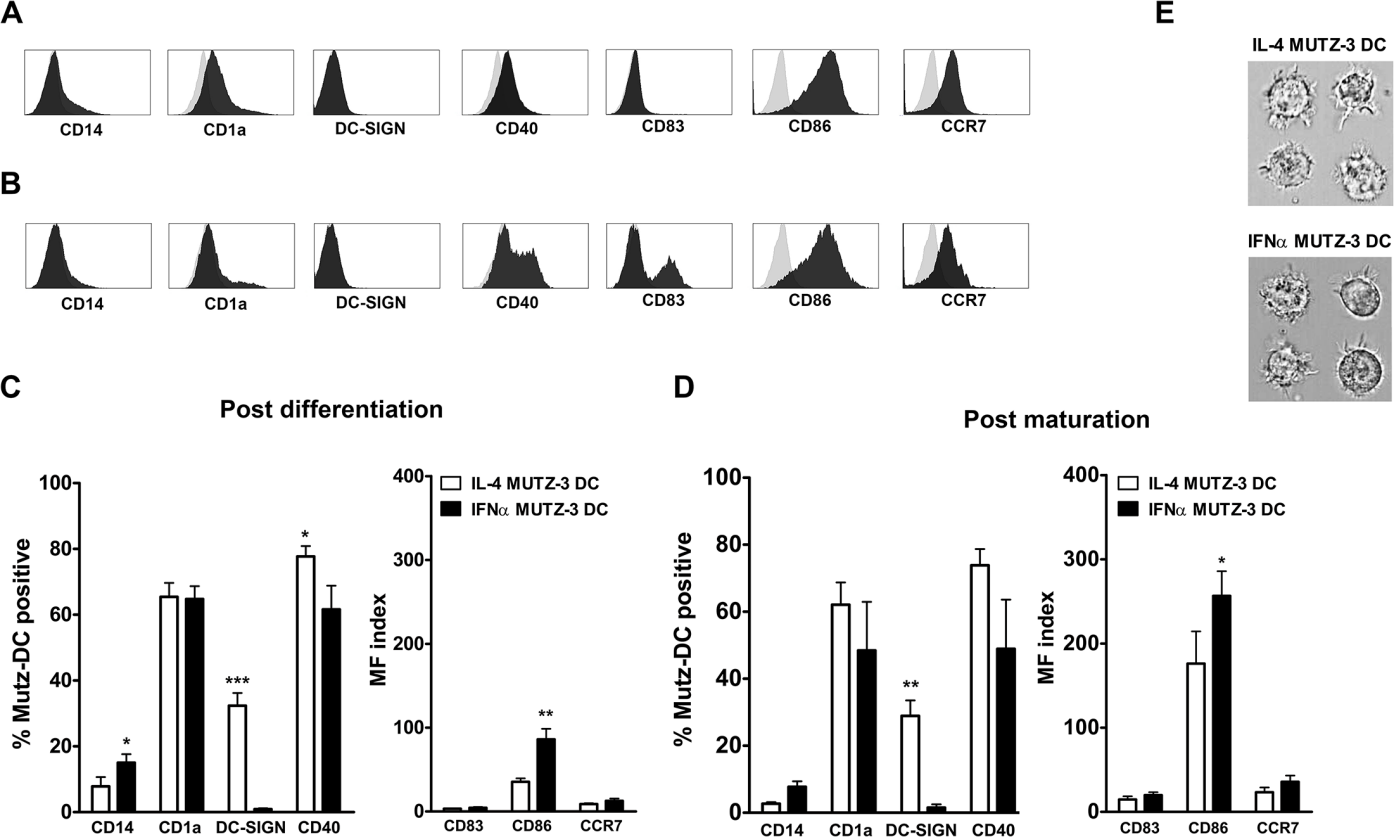
Phenotypic and morphologic analysis of IFNα- and IL-4 MUTZ-DC. (A) Representative example of the flow cytometric analysis of cell surface antigen expression profiles on IFNα MUTZ-DC following 3 days of differentiation and (B) following 24 hours of maturation. Represented are the isotype controls (grey) for each antigen (black). (C) Quantification of the flow cytometric analysis of DC-associated protein expression on IL-4 MUTZ-DC (white bars) and IFNα MUTZ-DC (black bars), directly following differentiation (C; n = 10) or after 24 hours of maturation (D; n = 4). The bars represent the mean values with the standard error of the mean (SEM). (E) Morphologic analysis of IL-4 (upper images) and IFNα MUTZ-DC (lower images), using the bright field channel of the ImageStream (magnification 60x). * p ≤ 0.05, ** p ≤ 0.01, *** p ≤ 0.001.

MUTZ-3 progenitors cells were cultured for 3 days to generate either IL-4, or IFNα MUTZ-DC. The yield of both MUTZ-DC types were very similar, ~53% of the initial progenitor cell number. Directly following differentiation, we analyzed the expression levels of common DC-associated cell surface antigens on IFNα MUTZ-DC (Fig 1A and 1C, and S1 Fig,) and IL-4 MUTZ-DC (Fig 1C, S1 and S3 Figs) using flow cytometry. Similarly to reports on IFNα MoDC [12], a significantly higher percentage of immature IFNα MUTZ-DC expressed CD14, the co-stimulatory molecule CD86, and reduced CD40 expression levels, as compared to IL-4 MUTZ-DC. Whereas IL-4 MUTZ-DC did express DC-SIGN (CD209), no cell surface expression of DC-SIGN was detected on IFNα MUTZ-DC (Fig 1A and 1C). Next, we analyzed the expression of these markers after inducing maturation for 24 hours, using a cytokine cocktail consisting of IL-1β, PGE-2 and TNFα (Fig 1B and 1D and S2 and S3 Figs). DC-SIGN was absent in mature (and immature, Fig 1C) IFNα MUTZ-DC, and CD1a was moderately up-regulated as compared to expression levels in mature IL-4 MUTZ-DC.In order to determine whether IFNα MUTZ-DC displayed typical morphological features of DC, we analyzed the appearance of both DC types using the ImageStream (bright field; Fig 1E). Both MUTZ-DC types displayed a clear and typical DC morphology, i.e. large cells with protruding dendrites, see Fig 1E.

### IFNα MUTZ-DC internalize exogenous antigen

**Fig 2:**
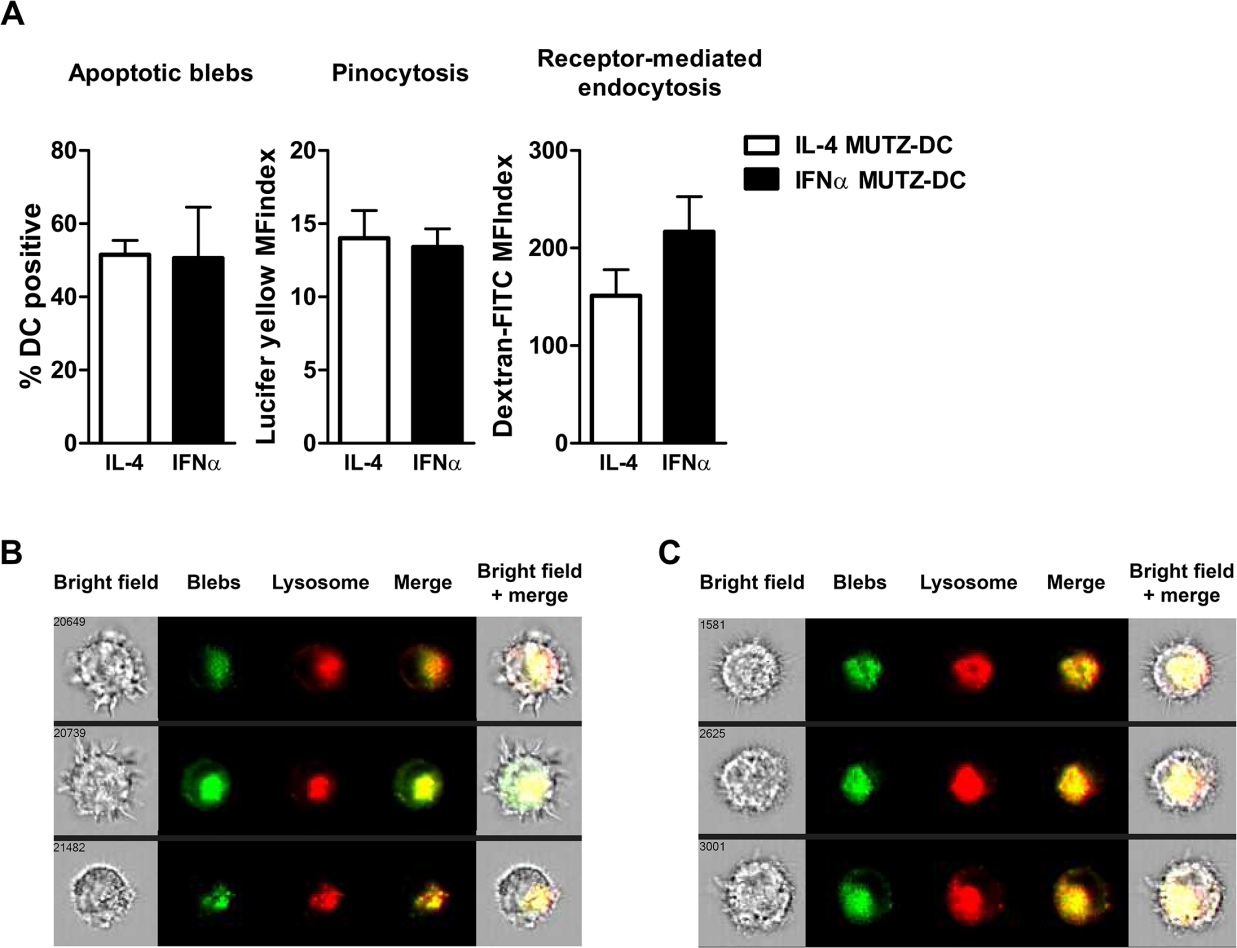
Phagocytic capacity of IFNα- and IL-4 MUTZ-DC. A) The capacity of DC to ingest exogenous antigen was analyzed and quantified using flow cytometry. Fluorescently labeled immature IL-4 (white bars) or IFNα MUTZ-DC (black bars) were loaded overnight with differentially labeled blebs, after which the percentage of double positive cells were quantified as a measure of uptake (A, Apoptotic blebs; n = 3). Pinocytosis and receptor-mediated endocytosis was assessed by culturing immature MUTZ-DC in the presence of Lucifer yellow (A, Pinocytosis; n = 3) or dextran-FITC (A, Receptor-mediated endocytosis; n = 3) for 1 hour, after the which the mean fluorescence intensity index (MFindex) was determined using flow cytometry. All bar graphs show the mean and SEM. (B) IL-4 MUTZ-DC and (C) IFNα MUTZ-DC were labeled with CFSE (not shown), co-cultured overnight with PKH26-labeled blebs (green), and labeled with LysoTracker (red). Next the co-localization of all fluorescent labels was analyzed using the ImageStream.

As the uptake of exogenous antigen is a hallmark of DC function and a pre-requisite for (cross-)presentation, we analyzed the ability of both MUTZ-DC types to ingest apoptotic blebs [21], as well as their pinocytotic capacity and lectin receptor-mediated endocytosis (Fig 2A). IL-4 and IFNα MUTZ-DC showed an equivalent capacity to ingest apoptotic blebs (Fig 2A; Apoptotic blebs), after overnight culture. Pinocytosis, a vital function of DC by which soluble exogenous antigens located in the tissues can be screened and invading pathogens detected, was analyzed by culturing MUTZ-DC in the presence of Lucifer yellow. IL-4 and IFNα MUTZ-DC also ingested soluble antigens via pinocytosis to a similar degree (Fig 2A; Pinocytosis). Moreover, both MUTZ-DC types were able to take up dextran via lectin receptor-mediated endocytosis to a similar degree (Fig 2A; Receptor-mediated endocytosis). To confirm that IFNα MUTZ-DC indeed internalized blebs, we differentially labeled IFNα MUTZ-DC (CFSE), the lysosomes (LysoTracker), and blebs (PKH26), and analyzed bleb/lysosome co-localization using the ImageStream following overnight loading. Both IL-4 MUTZ-DC (Fig 2B) and IFNα (Fig 2C) internalized blebs. Co-localization of ingested blebs with lysosomes indicates appropriate intracellular routing for subsequent antigen processing and presentation.

### IFNα MUTZ-DC migrate towards CCL21 and release pro-inflammatory cytokines

**Fig 3:**
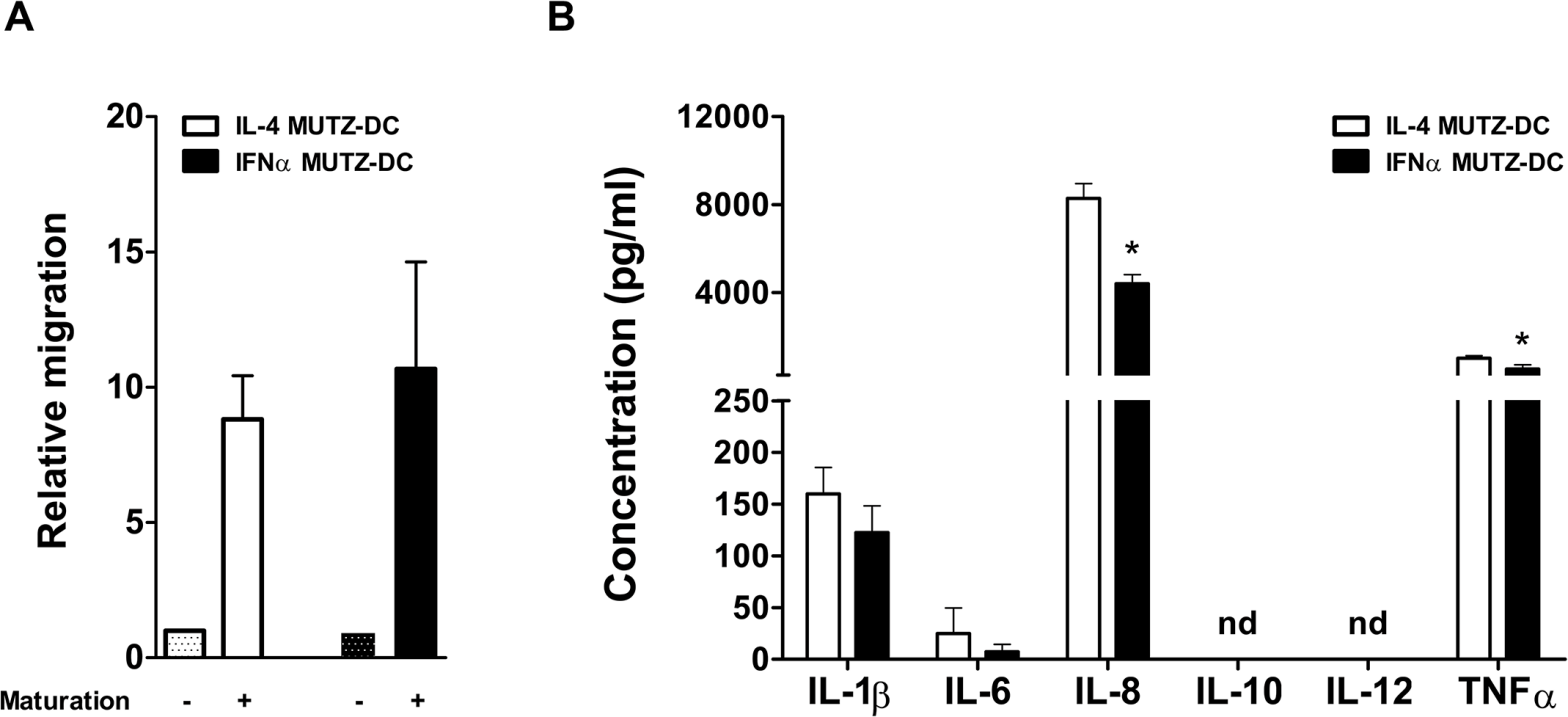
Migratory capacity and cytokine production by IFNα- and IL-4 MUTZ-DC. (A) The chemotactic migratory capacity of immature and mature IL-4 (white bars) or IFNα MUTZ-DC (black bars) was determined using a Transwell migration assay. The absolute number of actively migrated mature MUTZ-DC (maturation +) was measured relative to the migration of immature MUTZ-DC (maturation-). Shown is the mean relative migration and SEM (n = 3). (B). The cytokine production was assessed, by culturing MUTZ-DC with the CD40 ligand expressing cell line J558, in the presence of IFNγ. After 24 hours the cytokines produced by IL-4 (white bars) or IFNα MUTZ-DC (black bars) was determined. Shown is the mean cytokine production (pg/ml) and SEM (n = 3; nd: not detectable). * p ≤ 0.05.

DC entry into lymph nodes is critically dependent on their ability to migrate towards CCL21, a chemokine ligand for CCR7 [24]. We assessed the capacity of IFNα MUTZ-DC to migrate towards CCL21 in a trans-well migration assay. Indeed, mature (CCR7^+^) IFNα MUTZ-DC migrated towards CCL21 to a similar extent as IL-4 DC (Fig 3A).

After entering the lymph nodes, DC engage resident lymphocytes, which can subsequently become activated. The cytokines produced by DC are of importance, as they shape the initiated immune response(s). Upon CD40 ligation, in the presence of IFNγ, IFNα MUTZ-DC displayed a very similar cytokine release profile to IL-4 DC with relatively high levels of IL-8, IL1β and TNFα (Fig 3B).

### Allogeneic T cell priming and skewing by IFNα MUTZ-DC

**Fig 4:**
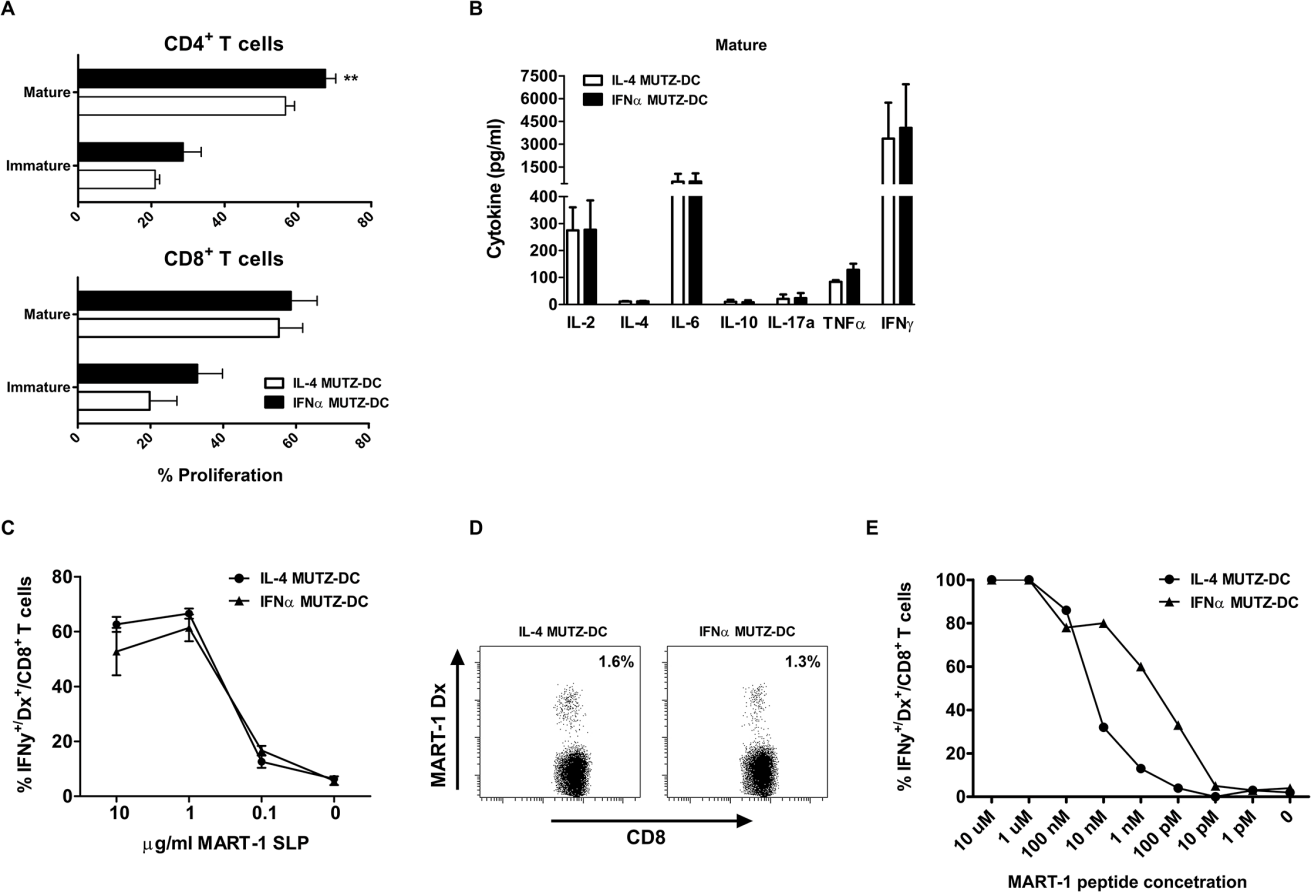
T cell proliferation and activation by IFNα- and IL-4 MUTZ-DC. (A) Immature or mature IL-4 (white bars) or IFNα MUTZ-DC (black bars) were cultured with CFSE-labeled allogeneic peripheral blood lymphocytes (PBL) for 6 days at a DC:PBL ratio of 1:5, after which the CFSE dilution was determined within the CD3^+^CD4^+^ and CD3^+^CD8^+^ T cell compartment, as a measure for proliferation (n = 6; ** p ≤ 0.01). The MLR supernatant was subsequently analyzed for the produced T cell cytokines (B; n = 3). Shown are the mean proliferation and cytokine production, with the corresponding SEM. (C) Immature IL-4 (circles) or IFNα (triangles) MUTZ-DC were loaded with a concentration titration (ranging from 0 μg/ml to 10 μg/ml) of a 25-mer MART-1 synthetic long peptide (SLP), in the presence of a cytokine maturation cocktail. After an overnight culture period, loaded MUTZ-DC were co-cultured with a MART CTL for 5 hours in the presence of a protein transport inhibitor, and the produced IFNγ was quantified using flow cytometry. (D) Mature IL-4 or IFNα MUTZ-DC were loaded exogenously with MART-1_26–35L_ peptide for 2 hours, after which they were co-cultured with naïve CD8^+^ T cells. Before every re-stimulation the percentage of MART-1 specific CD8^+^ T cells was determined using Dextramer (Dx) staining. Shown are representative examples of MART-1 specific CD8^+^ T cell outgrowth, following priming and one restimulation with either IL-4 or IFNα MUTZ-DC. (E) MART-1 specific CD8^+^ T cells were sorted and polyclonally expanded, after which they were rechallenged with MART-1_26–35L_ peptide-loaded JY cells. Intracellular IFNγ production was determined as a measure for activation.

Immature or mature MUTZ-DC were co-cultured with fluorescently labeled allogeneic peripheral blood lymphocytes (PBL) at a stimulator:responder ratio of 1:5. After 6 days the CD4^+^ and CD8^+^ T cell proliferation was analyzed (Fig 4A and S4 Fig). Like IL-4 DC, mature IFNα MUTZ-DC were able to prime both CD4^+^ and CD8^+^ T cells to a greater extent than immature IFNα MUTZ-DC (Fig 4A). Analysis of the produced T cell cytokines showed that very comparable levels of IL-2, IL-4, IL-6, IL-10, IL-17a, TNFα and IFNγ were produced upon priming by mature IL-4 or IFNα MUTZ-DC (Fig 4B).

### IFNα MUTZ-DC cross-present antigen and prime antigen-specific CD8^+^ T cells

As the presentation of exogenously acquired antigen in an HLA class I restricted fashion to CD8^+^ T cells is a key feature of DC, we analyzed the ability of both MUTZ-DC types to cross-present the MART-1_aa26-35_ immunodominant epitope from a 25-mer MART-1 synthetic long peptide (SLP, aa16-40L) to a specific CD8^+^ cytotoxic T cell line (MART-1 CTL). MUTZ-DC were loaded overnight with different concentrations of the MART-1 SLP in the presence of a maturation cytokine cocktail. After co-culture with the MART-1 CTL in the presence of a protein transport inhibitor, we analyzed the accumulation of intracellular IFNγ as a measure of antigen recognition. Both MUTZ-DC types were able to cross-present soluble antigen with similar efficiency (Fig 4C, and S5 Fig). Finally, we assessed the ability of IFNα MUTZ-DC to prime naïve CD8^+^ T cells. To this end, we loaded them with a MART-1_aa26-35L_ 9-mer peptide for 2 hours and co-cultured them with naïve HLA-A2 matched CD8^+^ T cells, after which the MART-1-specific T cell outgrowth was analyzed. IFNα, as well as IL-4 MUTZ-DC, were capable of priming antigen-specific CD8^+^ T cells (Fig 4D). Moreover, the specific CD8^+^ CTL primed by IFNα MUTZ-DC were functional, as they specifically produced IFNγ upon a re-challenge with the MART-1_aa26-35L_ peptide with intermediate functional avidity (Fig 4E).

## Discussion

MoDC have been used to study DC function and for (anti-cancer) DC vaccination purposes. MoDC can be differentiated from their CD14^+^ monocyte precursor, which is present in the blood at relatively high numbers. However, studying DC function *in vitro* often involves a frequent supply of (relatively) large numbers of monocytes, and thus requiring large volumes of blood. Alternatively, DC can be cultured from CD34^+^ hematopoietic progenitor cells, under the influence of c-kit-ligand, GM-CSF and TNFα [4], or IL-3 and IL-6 [5]. Cell line models provide a durable platform to perform mechanistic studies, allowing for genetic modification with minimal variability. As there is an increasing interest in studying IFNα DC and no such cell line model has been described to date, we assessed the possibility to generate IFNα MUTZ-DC from CD34^+^ MUTZ-3 progenitor cells. Since IL-4 MUTZ-DC is an established model for studying DC function in vitro [16], and since MUTZ-DC are derived from early CD34^+^ progenitors, in contrast to MoDC, we performed a head-to-head comparison with IL-4 MUTZ-DC in this study, as we deemed this a more meaningful comparison. Culturing MUTZ-3 progenitor cells in the presence of IFNα, GM-CSF and mitoxantrone induced their differentiation into cells morphologically resembling DC. Moreover, the cell surface expression of common DC differentiation and maturation molecules was very similar to the expression of IFNα MoDC [12]. Similar to IL-4 vs IFNα MoDC, IFNα MUTZ-DC had an increased expression of CD14 and CD86 post-differentiation. DC-SIGN was absent from IFNα MoDC in contrast to IL-4 MUTZ-DC, in line with its known regulation through IL-4, and negative regulation by IFNα [25]. IFNα MUTZ-DC displayed a remarkably similar antigen uptake efficiency compared to their MoDC counterpart [12]. Likewise, the relative cytokine production of IL-4 MUTZ-DC was identical as compared to IFNα MUTZ-DC upon CD40 ligation. Moreover, IFNα MUTZ-DC displayed DC functionality, as they were capable of actively migrating towards the lymph node homing chemokine CCL21, as well as inducing T cell proliferation in a mixed leukocyte reaction. An important function of DC is their ability to cross-present antigen, a mechanism by which exogenous antigen are presented to CD8^+^ T cells [26]. We showed that IFNα MUTZ-DC were able to cross-present a 25-mer synthetic long MART-1 peptide very efficiently, and could prime functional antigen-specific CD8^+^ T cells, showing that IFNα MUTZ-DC are functional DC, as described previously for IL-4 MUTZ-DC [14,19,20]. Whether either MUTZ-DC type is more potent in cross-presentation and-priming of cell-associated antigens, warrants further investigation in future studies.The *in vivo* relevance of IFNα is clear, especially during viral infection, where large amounts of IFNα are produced. IFNα is mainly produced by plasmacytoid DC [27], and has been shown to induce the differentiation and maturation of myeloid DC [28,29]. Being intracellular pathogens, increasing the ability to cross-present antigen following viral infection is beneficial. This mechanism can be exploited to increase cross-presentation in cancer patients, by differentiating DC in the presence of IFNα. Indeed, IFNα treatment proved beneficial in chronic myeloid leukemia, based on increased differentiation of CML cells to DC [30], and increased numbers of tumor-specific CD8^+^ T cells [31], which could also be the result of the increased differentiation of DC under influence of IFNα. Whether either MUTZ-DC type is more potent in cross-presenting and–priming CD8^+^ T cells after phagocytosis of cell-associated antigen, or indeed from other sources of TAA, warrants more in-depth investigation in follow-up studies and may be of critical importance when IFNα MUTZ-DC are to be exploited in a clinical setting.

In conclusion, next to the previously described use as a model for studying IL-4 DC [14,20], we now show that the MUTZ-3 cell line is instrumental as a sustainable IFNα-induced human DC differentiation model. IFNα MUTZ-DC harbor all major functional characteristics described for DC, such as the uptake of exogenous antigen, active chemotactic migration, and importantly, the ability to cross-present antigen and prime functional antigen-specific CD8^+^ T cells. The model described here, is highly supportive to further study the application of IFNα DC *in vitro* and *in vivo*.

## Supporting Information

S1 Fig IL-4 and IFNα MUTZ-DC immature phenotype. The mean fluorescent index (MFI) values of the flow cytometric analysis of IL-4 and IFNα MUTZ-DC after differentiation.
(TIF)

S2 Fig IL-4 and IFNα MUTZ-DC mature phenotype. The mean fluorescent index (MFI) values of the flow cytometric analysis of IL-4 and IFNα MUTZ-DC after maturation.
(TIF)

S3 Fig Representative flow cytometry histograms. Histograms of flow cytometric analysis of IL-4 MUTZ-DC after differentiation (top) and maturation (bottom).
(TIF)

S4 Fig Representative examples of T cell proliferation by flow cytometry. CD3^+^7AAD^-^ cells where gated from the viable lymphocytes, and the CFSE dilution of CD4^+^ and CD8^+^ T cells was analyzed as a measure for T cell proliferation, after 5 days of co-culture with either IL-4 or IFNα MUTZ-DC in an MLR.
(TIF)

S5 Fig Cross-presentation by IL-4 and IFNα MUTZ-DC. IL-4 or IFNα MUTZ-DC were loaded overnight with different concentrations of MART-1 SLP in the presence of a maturation cocktail. Loaded MUTZ-DC were co-cultured with a MART CTL for 5 hours in the presence of a protein transport inhibitor, after which the accumulated IFNγ was determined as a measure for CTL activation, as a consequence of cross-presentation of the MART-1 SLP.
(TIF)
