# Supplementary figures and images for: A Human Cell Line Model for Interferon-α Driven Dendritic Cell Differentiation
Source: PLoS One. 2015 Aug 7;10(8):e0135219. doi: 10.1371/journal.pone.0135219 (PMC4529224; doi:10.1371/journal.pone.0135219)

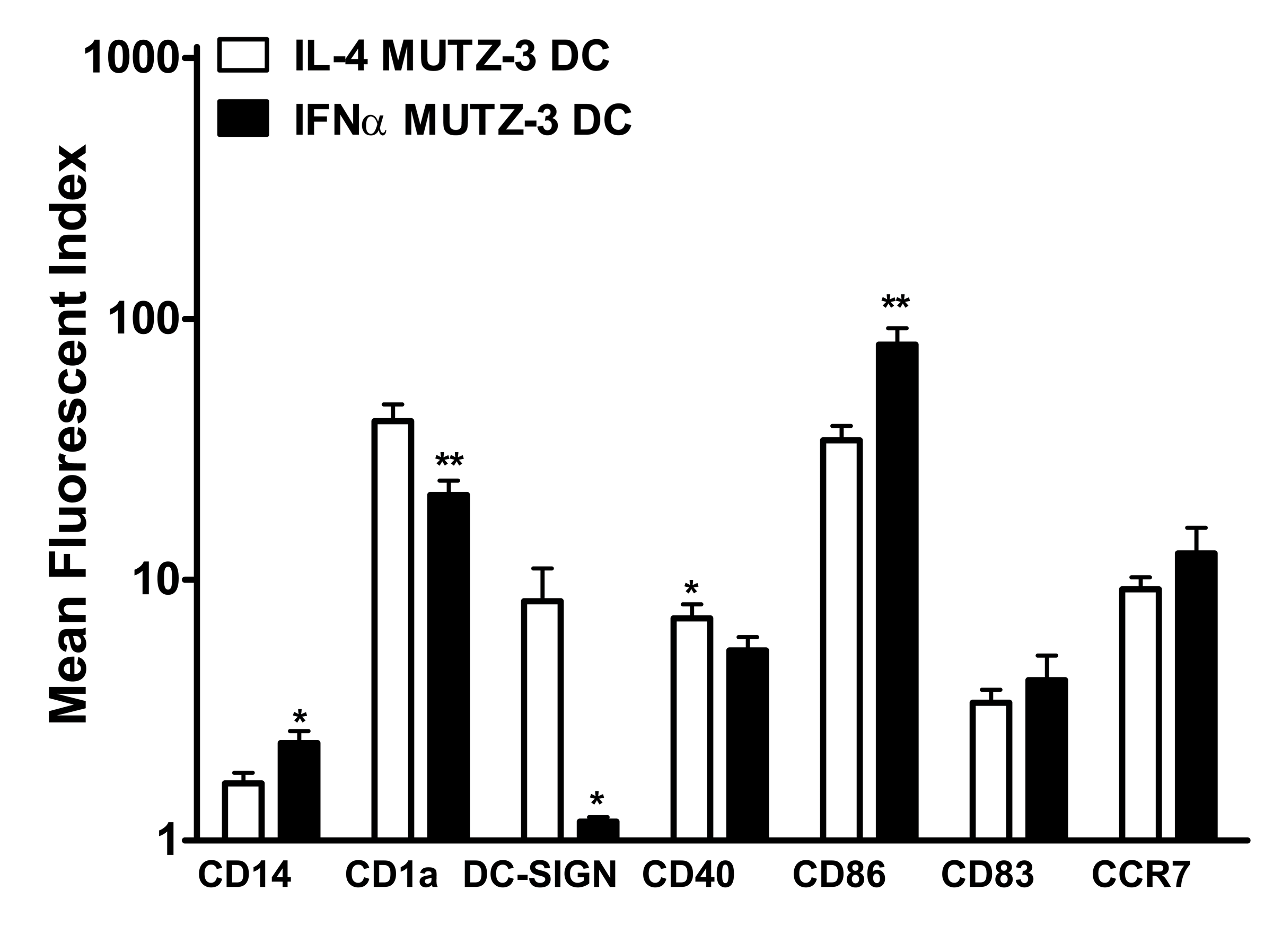

Supplement: S1 Fig — The mean fluorescent index (MFI) values of the flow cytometric analysis of IL-4 and IFNα MUTZ-DC after differentiation. (TIF) [file pone.0135219.s001.tif]

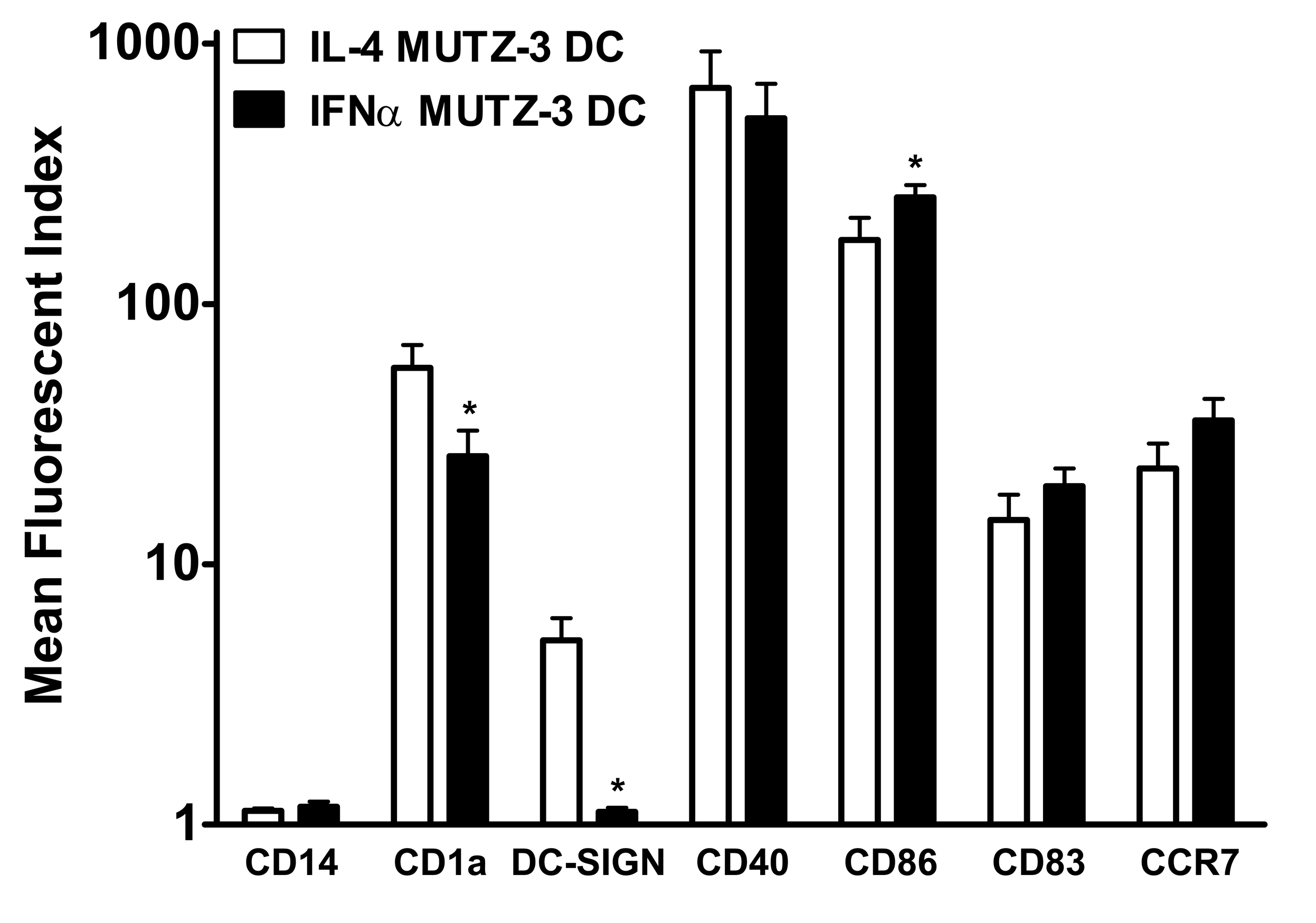

Supplement: S2 Fig — The mean fluorescent index (MFI) values of the flow cytometric analysis of IL-4 and IFNα MUTZ-DC after maturation. (TIF) [file pone.0135219.s002.tif]

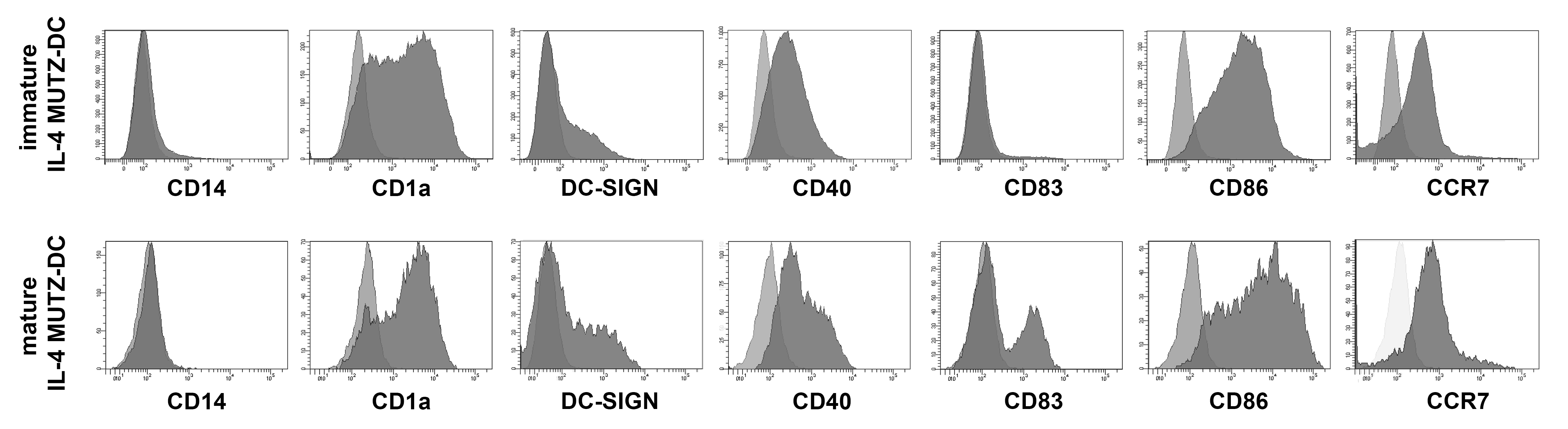

Supplement: S3 Fig — Histograms of flow cytometric analysis of IL-4 MUTZ-DC after differentiation (top) and maturation (bottom). (TIF) [file pone.0135219.s003.tif]

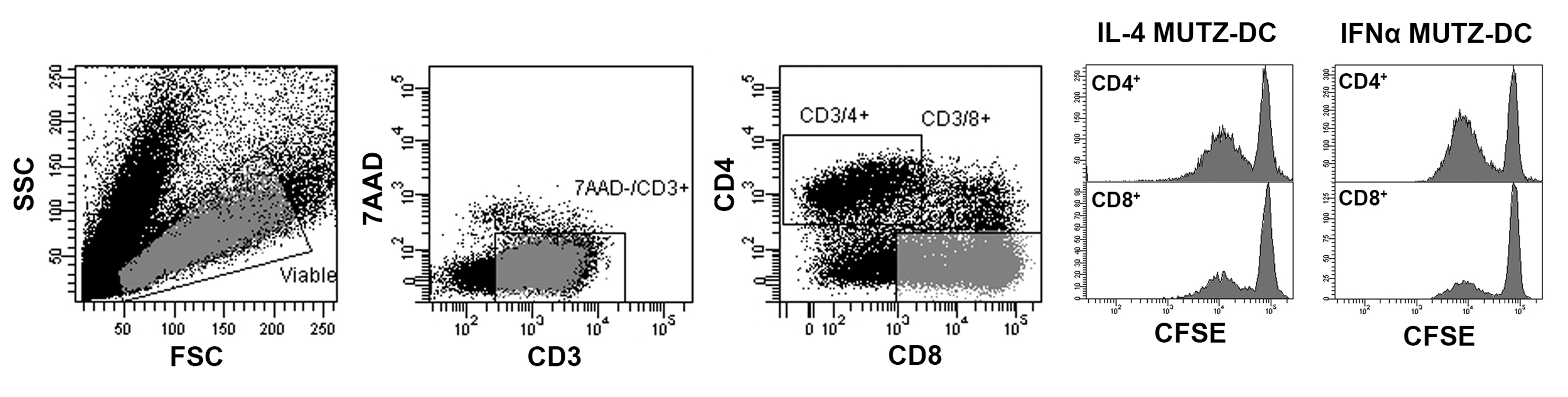

Supplement: S4 Fig — CD3+7AAD- cells where gated from the viable lymphocytes, and the CFSE dilution of CD4+ and CD8+ T cells was analyzed as a measure for T cell proliferation, after 5 days of co-culture with either IL-4 or IFNα MUTZ-DC in an MLR. (TIF) [file pone.0135219.s004.tif]

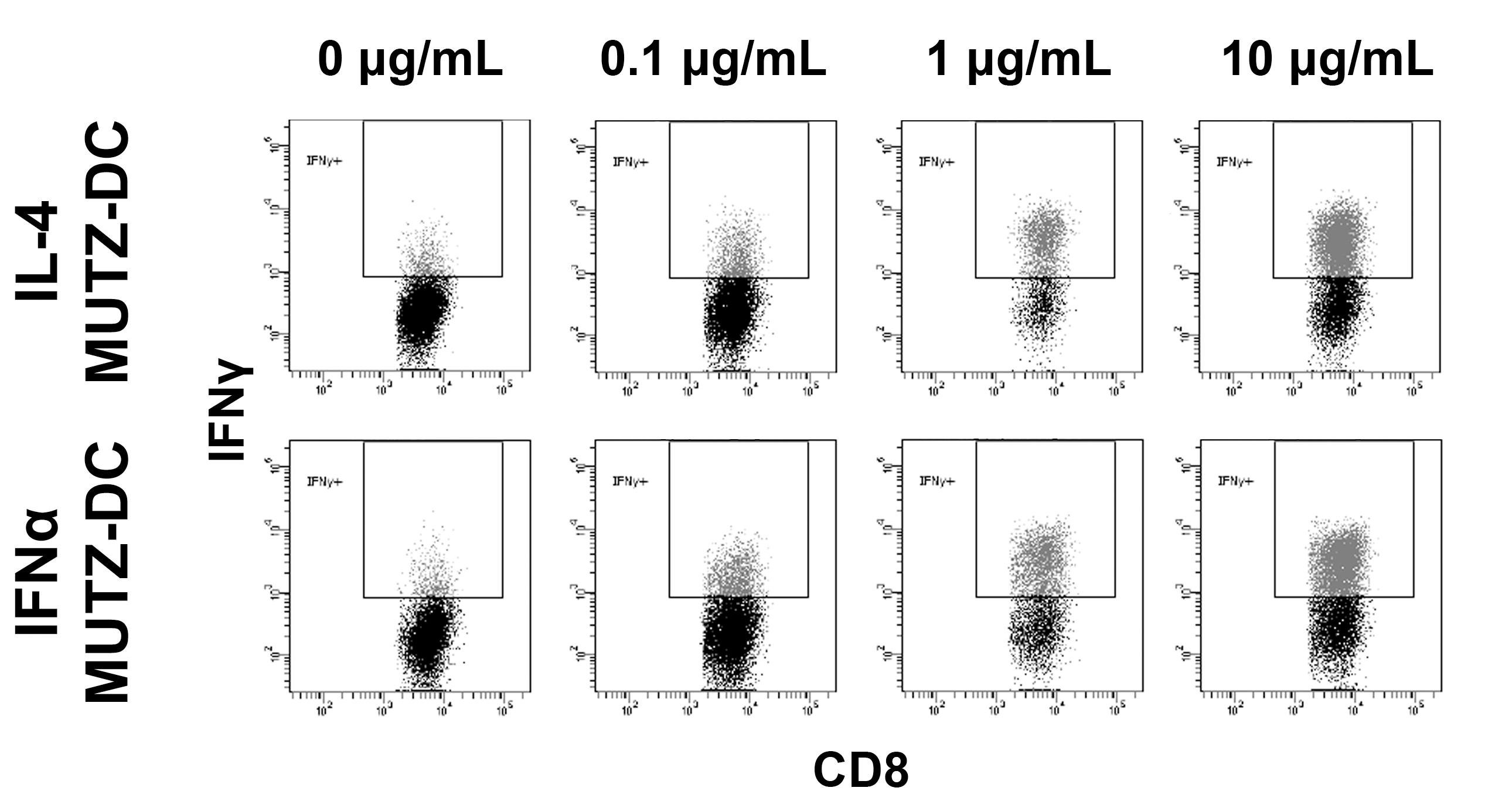

Supplement: S5 Fig — IL-4 or IFNα MUTZ-DC were loaded overnight with different concentrations of MART-1 SLP in the presence of a maturation cocktail. Loaded MUTZ-DC were co-cultured with a MART CTL for 5 hours in the presence of a protein transport inhibitor, after which the accumulated IFNγ was determined as a measure for CTL activation, as a consequence of cross-presentation of the MART-1 SLP. (TIF) [file pone.0135219.s005.tif]
